# Indoor Localization Based on VIO System and Three-Dimensional Map Matching

**DOI:** 10.3390/s20102790

**Published:** 2020-05-14

**Authors:** Jitong Zhang, Mingrong Ren, Pu Wang, Juan Meng, Yuman Mu

**Affiliations:** 1 College of Automation, Faculty of Information Technology, Beijing University of Technology, Beijing 100124, China; a17812102961@163.com (J.Z.); wangpu@bjut.edu.cn (P.W.); S201761125@emails.bjut.edu.cn (J.M.); mu3218509460@163.com (Y.M.); 2 Engineering Research Center of Digital Community, Ministry of Education, Beijing 100124, China; 3 Beijing Key Laboratory of Computational Intelligence and Intelligent Systems, Beijing 100124, China

**Keywords:** visual inertial odometry, indoor localization, conditional random field, map matching, three-dimensional

## Abstract

High-precision indoor localization plays a vital role in various places. In recent years, visual inertial odometry (VIO) system has achieved outstanding progress in the field of indoor localization. However, it is easily affected by poor lighting and featureless environments. For this problem, we propose an indoor localization algorithm based on VIO system and three-dimensional (3D) map matching. The 3D map matching is to add height matching on the basis of previous two-dimensional (2D) matching so that the algorithm has more universal applicability. Firstly, the conditional random field model is established. Secondly, an indoor three-dimensional digital map is used as a priori information. Thirdly, the pose and position information output by the VIO system are used as the observation information of the conditional random field (CRF). Finally, the optimal states sequence is obtained and employed as the feedback information to correct the trajectory of VIO system. Experimental results show that our algorithm can effectively improve the positioning accuracy of VIO system in the indoor area of poor lighting and featureless.

## 1. Introduction

With the development of society, high-precision positioning has become indispensable in many places. In the unobstructed outdoor environment, we can use the high-precision Global Positioning System (GPS) [1,2,3] for positioning. However, in the indoor environment, the GPS positioning performance is poor due to the GPS signals being blocked and reflected by the building. Therefore, traditional indoor positioning mostly uses inertial measurement units [4,5], Wi-Fi [6,7], ultrawideband (UWB) [8], and Bluetooth [9,10] for positioning. However, unlike GPS, none of these methods have been shown to be most suitable for the majority of applications. When inertial sensors are used for positioning, error correction algorithms must be used to assist the positioning, such as zero velocity update algorithm [11] and gyro drift measurement technology [12]. Nevertheless, inertial sensors will still produce large cumulative error after a period of time. The Wi-Fi positioning and UWB technology need to set up the corresponding infrastructure indoors, and the signal is also very susceptible to interference, resulting in large deviations in positioning. For Bluetooth indoor positioning technology, Bluetooth Low Energy (BLE) technology has been used in recent years. In a study [9], the author introduces a wireless BLE Single Input Multiple Output (SIMO) system. The algorithm simplifies the deployment procedures in corridors. In another study [10], a Pedestrian Dead Reckoning (PDR) based indoor positioning system with iBeacon calibrations is proposed. The system can be implemented in resource-limited embedded device. However, BLE technology requires multiple iBeacon devices to be installed indoors, and the Bluetooth signal is also vulnerable to interference leading to inaccurate positioning. In addition, the later device maintenance is also a problem. 

Visual inertial odometry is a well-known technology that uses cameras and inertial measurement unit (IMU) to estimate the position and motion trajectory of pedestrians or robots. It combines the information of vision and IMU to obtain higher accuracy location information than single vision and single IMU [13]. It can be used on micro aerial vehicles to achieve positioning and navigation [14]. It can also be used to implement the robot simultaneous localization and mapping (SLAM) function [15]. The use of VIO technology for indoor positioning has attracted the attention of many scholars in recent years [16,17,18]. However, one of the drawbacks of VIO indoor positioning is that in the place where indoor poor lighting and featureless environments, enough feature points cannot be extracted from the images taken by camera. Therefore, effective matching between two images cannot be carried out and accurate positioning cannot be obtained. Study [19] proposed to extract the point and line features in the image. Compared with point features, lines provide significantly more geometrical structure information on the environment. This algorithm has a certain effect, but in an environment with few texture features, such as white wall, the effect is not obvious. So, we considered using other algorithm to correct the positioning accuracy of VIO in these areas.

Many researchers have been paying attention to the application of map matching algorithm in indoor localization. The map matching algorithm proposed in [20,21] adopts particle filtering algorithm. The advantage of particle filtering algorithm is that it can be used in nonlinear non-Gaussian systems. But the disadvantage is also obvious, the computational workload of particle filtering is related to the number of particles. Once the number of particles increases, the computational workload will also increase rapidly, which will bring great computational burden. Studies [22,23] adopt a map matching algorithm based on hidden Markov model (HMM), but this method assumes that the observations are independent of each other, which is not suitable for our system. Recently, with the research of conditional random field model algorithm, it has made significant progress in map matching. In 2014, Xiao and Zhuoling from the University of Oxford applied the conditional random field (CRF) model to map matching to improve the inertial localization [24]. Study [25] has proved that compared with other algorithms, the CRF model can capture a variety of constraints relationships between observations, with more universal applicability and higher accuracy. In 2016, Study [26] proposes an offline map matching algorithm designed for indoor localization systems based on CRF. The algorithm uses loose coupling between the localization system and the proposed map matching technique. In 2017, we combined Microelectromechanical system (MEMS) with 2D map matching algorithm for indoor positioning and achieved good results [27].

Therefore, we propose an indoor positioning algorithm that combines 3D map matching based on a CRF and the VIO system, and uses the optimal matching states sequence to correct the VIO trajectory. First of all, we need to obtain the high-precision indoor map and digitize it to get a usable digital map, and then pre-process the states of the map. The coordinates of each point are vectorized and stored by using evenly spaced points to cover the whole indoor area. In order to match the indoor height information, we also set the states on the stairs. Finally, we remove the states of those unreachable areas based on the structure of the indoor map, which can reduce mismatches and the trajectories pass through walls. Our main contributions as follows:

- An algorithm based on 3D map matching and VIO system is proposed for indoor localization.
- The conditional random field model of 3D map matching algorithm is established.
- According to the different walking characteristics of pedestrian in plane and stair stage, different state definition methods are adopted.
- In order to verify the reliability of our algorithm, we have carried out multiple experiments and compared the performance of our system with only single VIO system. Experiments show that our algorithm can achieve more accurate and robust positioning effects than a single VIO system in indoor environment with insufficient light and texture.

The remainder of this paper is organized as follows. In Section 2, we will introduce the system overview. In Section 3, we will introduce the map matching algorithm based on CRF model. Section 4 gives the details and results of our experiment. Finally, Section 5 is the summary of the full text and the prospect of the future work.

## 2. System Overview

**Figure 1:**
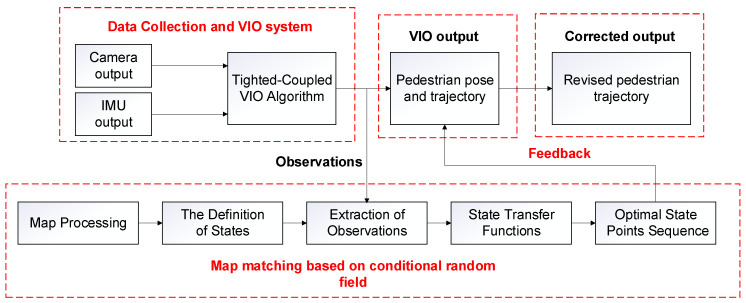
The framework of the proposed algorithm.

The overall framework of our approach is shown in Figure 1. The system is mainly divided into the following three parts: the data collection and VIO system, map matching based on conditional random field model and the feedback. The first part is the data collection and VIO system, we use IMU and camera to collect data. The IMU consists of a three-axis gyroscope and an accelerometer, which can collect acceleration and angular velocity information at each moment. The camera can capture the image information at each time. Then we transmit the collected data to the computer for the tightly-coupled VIO algorithm, which can obtain the pedestrian trajectory and pose at each moment. The second part is the map matching based on conditional random field, which uses the conditional random field to fuse the pose of the pedestrian at each moment obtained by the VIO system with the data of the map matching system to obtain an accurate matching trajectory. The third part is the feedback part. By taking the matching result as the feedback, the pose of the pedestrian at each moment of the VIO system are corrected to obtain the final corrected position trajectory.

**Figure 2:**
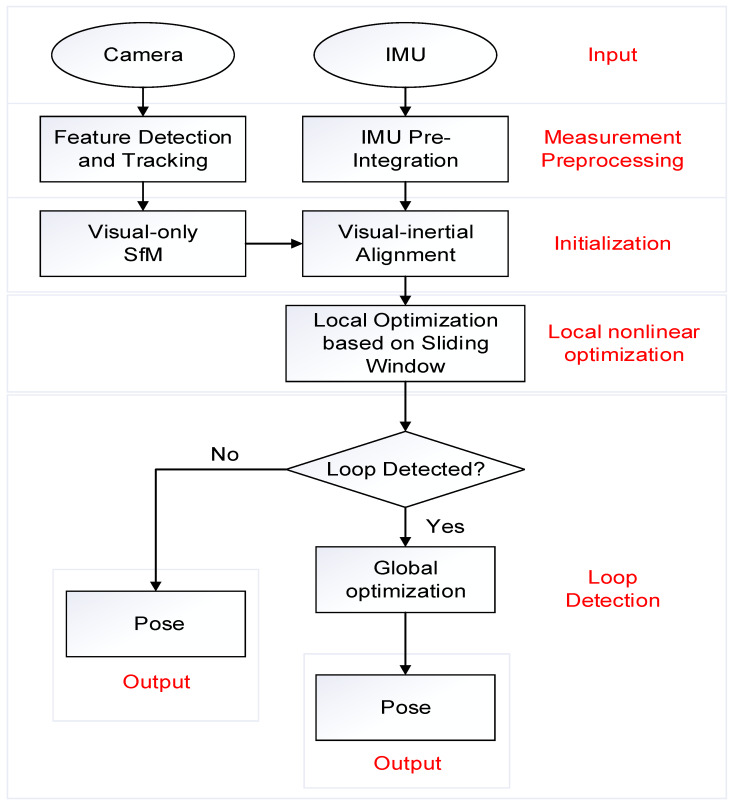
The flow chart of the visual inertial odometry (VIO) algorithm.

The VIO system we use is the vins-mono open source system proposed by Tong Qin et al. in 2017 [28]. It is the state-of-the-art tightly-coupled VIO system based on nonlinear optimization. The whole approach mainly includes four parts: IMU pre-integration, initialization, local nonlinear optimization, and loop detection. Figure 2 is the VIO algorithm flow chart. Firstly, the camera and IMU are used to collect data, extract feature points from the pictures taken by the camera and track the feature points, and perform IMU pre-integration on the data collected by the IMU. Secondly, Structure from Motion (SfM) algorithm is used to perform visual pose estimation, and then it is aligned with IMU pre-integration to solve the initialization parameters. After that, local nonlinear optimization is performed to optimize the state vectors in a sliding window. Finally, the loop detection will be judged. If there is no loop occurs, the pose will be output directly, otherwise, the global optimization will be performed, and then the pose will be output.

## 3. Indoor 3D Map Matching Algorithm Based on CRF

We establish conditional random field model of indoor 3D map, and then estimate the optimal states sequence through observation information and model parameters. The optimal states sequence is used to correct the VIO trajectory.

### 3.1. Linear-Chain Conditional Random Field

The conditional random field algorithm is a conditional probability distribution model of output random variables when given a set of input random variables [29]. A special form of CRF is the random field model of linear chain. In this model, the output variables are modeled as a sequence, when given the input $x=\left(x_{1},x_{2}\cdots x_{n}\right)$, the probability of the output $y=\left(y_{1},y_{2},\cdots y_{n}\right)$ has the following form:

(1) $$p\left(y|x\right)=\frac{1}{Z\left(x\right)}exp\left(\overset{k}{\underset{k=1}{\sum }}w_{k}f_{k}\left(y,x\right)\right).$$

where $f_{k}$ is local state transfer function and $w_{k}$ is corresponding weight. Z(x) is the normalization factor and its expression is:

(2) $$Z\left(x\right)=\underset{y}{\sum }exp\overset{K}{\underset{k=1}{\sum }}w_{k}f_{k}\left(y,x\right).$$

We can use w and F(y,x) to represent the weight vector and the global state transfer function vector.

(3) $$\{\begin{array}{l} w={\left(w_{1},w_{2},\cdots ,w_{K}\right)}^{T}\\ F\left(y,x\right)={\left(f_{1}\left(y,x\right),f_{2}\left(y,x\right),\cdots f_{K}\left(y,x\right)\right)}^{T} \end{array}.$$

Therefore, Equation (1) can be rewritten as:

(4) $$p\left(y|x\right)=\frac{exp\left(w\cdot F\left(y,x\right)\right)}{Z_{w}\left(x\right)}.$$

where:

(5) $$Z_{w}\left(x\right)=\underset{y}{\sum }exp\left(w\cdot F\left(y,x\right)\right).$$

### 3.2. Map Pre-Processing

**Figure 3:**
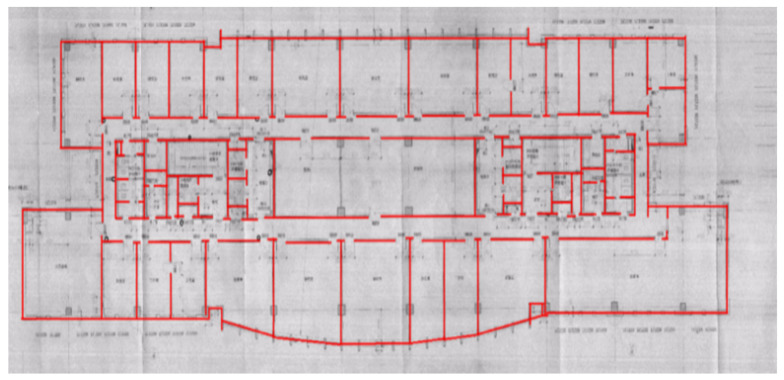
Digital format map.

We need to process the indoor map before the experiment to obtain the discrete state of each moment. The indoor map is image format, which is not suitable for direct processing, so the software MapInfo is first used to convert the indoor maps from image format into digital format. The digital information of the indoor structure is obtained according to the calibration, projection, and description, and the digital information format is the endpoint coordinates of each line segment. The experiment site and map used are science building of Beijing University of Technology. The processed digital map is shown in Figure 3.

### 3.3. The Definition of States

We use equal-sized square cells to divide the map and the vertices of each cell are the possible hidden states. The side length of each square cell is set as 0.8 m. At the same time, in order to achieve height matching, we use cuboids of equal size to divide the stair area between two floors. The height of cuboids is 0.16 m, which is equal to the height of a step, and the length and width of cuboids are 0.8 m, which is equal to the side length of the cell used before. The vertices of each cuboid are the possible hidden states and each state stores the corresponding coordinate information in the map coordinate system.

**Figure 4:**
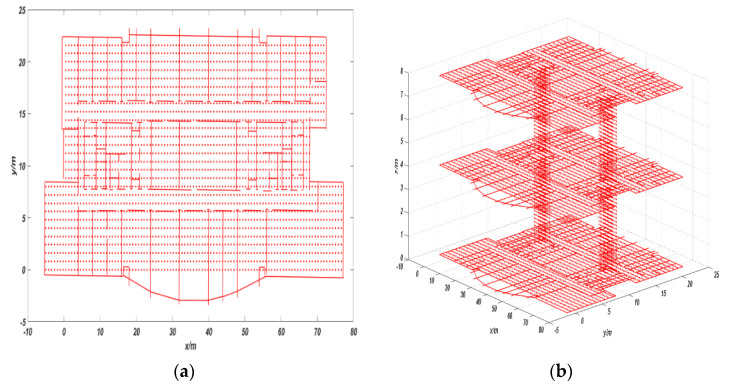
(**a**) Two-dimensional map with states and (**b**) three-dimensional map with states.

In order to improve the matching accuracy, we removed the states of those unreachable areas according to the specific situation of the indoor map. For example, if the vertices of the cells and cuboids in the map are too close to indoor impassable areas (such as walls, pillars, etc.), we will delete this state. Meanwhile, there are some constraints between the propagation of states. When the edge between two cell vertices passes through a previously unreachable area, the state cannot transfer directly. The processed map with states is shown in Figure 4, where, the red points represent the states, and the red lines represent the indoor structure.

### 3.4. Extraction of Observations

Pedestrians have different state characteristics when walking on the plane and walking on the stairs. Therefore, the extraction of observations should be divided into different cases. In the corridor 2D plane phase, we decided to extract the VIO output position information at the current moment as the observations when the pedestrian walking distance reached a fixed threshold. The threshold is set at 0.8 m, which is equal to the minimum distance between the two states. Its process is shown in Algorithm 1.

**Algorithm 1.** Extraction of Observations.

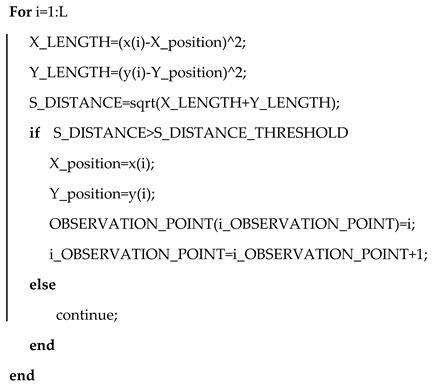



In the stairs phase, the change in height is involved. At the same time, when pedestrian walk on the stairs, each step will stay on the steps for a period of time, during which the speed is 0. So, we set that when the pedestrian’s height value changes at 0.16 m and the speed is 0, we extract the VIO output at the current moment as the observation. 0.16 m is the height of the cuboid set before, which is also the height of a step.

(6) $$\{\begin{array}{c} x_{t}\left(z\right)-x_{t-1}\left(z\right)\geq 0.16\\ v_{t}=0 \end{array}$$

### 3.5. Establishment of State Transfer Function

**Figure 5:**
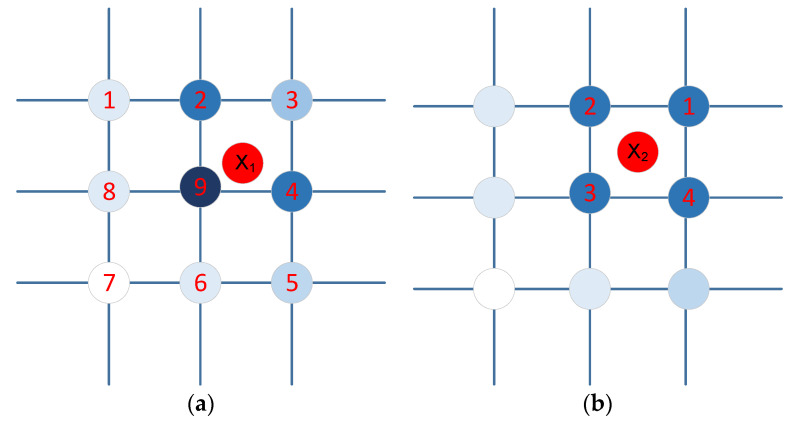
Match selection between observation values and state points: (**a**) based on distance and (**b**) based on heading.

The state transfer functions represent the support degree of the observation for the transition between consecutive states. When an observation matches a state point, an obvious rule is that the smaller the distance to this state point, the greater the probability of this observation at this state point, and vice versa. As shown in Figure 5a, the red point $X_{1}$ is an observation value output by VIO, and the remaining points are the state points on the map. During the process of $X_{1}$ matching the state points, the dark color state points in the figure below indicate the points with higher matching probability, while the light color state points in the figure below indicate the points with a low matching probability. Another factor that affects matching is the heading information. As shown in Figure 5b, the four state points (1,2,3,4) around the observation value $X_{2}$ are the same distance from $X_{2}$. At this time, we can use the information of $X_{2}$ heading information. The specific method is shown in Table 1.

Therefore, the state transfer function can be established based on distance and orientation. In the plane phase, it is the matching of two-dimensional state points. We use the distance and orientation between the pose information output by the VIO system at the current moment and the pose information output by the VIO system at the previous moment as two features to establish state transfer functions.

The first state transfer function is established based on the position information of the VIO system at the current moment and the previous moment:

(7) $$f_{1}\left(y_{t},y_{t-1},x_{t}^{d}\right)=Ln\frac{1}{\sigma_{d}\sqrt{2\pi }}-\frac{{\left(x_{t}^{d}-d\left(y_{t-1},y_{t}\right)\right)}^{2}}{2\sigma_{d}^{2}},$$

where $x_{t}^{d}$ is the Euclidean distance between two consecutive observations, $d\left(y_{t-1},y_{t}\right)$ is the Euclidean distance between two consecutive state points, and $\sigma_{d}^{2}$ is the variance of the distance in the observation data.

In the second state transfer function, we select the orientation information between the position output by the current VIO system and the position by the previous VIO system as a feature to establish state transfer function:

(8) $$f_{2}\left(y_{t},y_{t-1},x_{t}^{\theta }\right)=Ln\frac{1}{\sigma_{\theta }\sqrt{2\pi }}-\frac{{\left(x_{t}^{\theta }-\theta \left(y_{t-1},y_{t}\right)\right)}^{2}}{2\sigma_{\theta }^{2}},$$

where $x_{t}^{\theta }$ is the orientation of two consecutive observations, $\theta \left(y_{t-1},y_{t}\right)$ is the orientation between two consecutive state points, and $\sigma_{\theta }^{2}$ is the variance of the orientation in the observation data.

In the stair phase, we need to add the one-dimensional state points matching for the height. Each step of the pedestrian will correspond to a real height value. We can use the error between the height value matched by each step of the pedestrian and the height value output by VIO as a height matched feature to establish the state transfer functions:

(9) $$f_{3}\left(y_{t},x_{t},M_{t}\left(x,y\right)\right)=Ln\frac{1}{\sigma_{e}\sqrt{2\pi }}-\frac{{\left(\left(y_{t}-x_{t}\right)-M_{t}\left(x,y\right)\right)}^{2}}{2\sigma_{e}^{2}},$$

where $y_{t}$ is the height value of best match at time *t*, $x_{t}$ is the height value of the VIO output at time *t*, $\delta_{e}^{2}$ is the height error variance, and $M_{t}\left(x,y\right)$ is the average of all the errors before time *t*.

Therefore, the Equation (4) can be written as:

(10) $$p\left(y|x\right)=\frac{1}{Z_{w}\left(x\right)}exp\left(w_{1}f_{1}\left(y_{t},y_{t-1},x_{t}^{d}\right)+w_{2}f_{2}\left(y_{t},y_{t-1},x_{t}^{\theta }\right)+w_{3}f_{3}\left(y_{t},x_{t},M_{t}\left(x,y\right)\right)\right).$$

### 3.6. Optimal State Points Sequence

For the solution of Equation (10), we adopt the Viterbi algorithm [30]. When the observation value sequence is given, Viterbi algorithm can dynamically solve the optimal state points sequence that is most likely to produce the currently given observation value sequence. The solution steps of Viterbi algorithm are as follows:

(1) Initialization: Compute the non-normalized probability of the first position for all states, where m is the number of states.

(11) $$\delta_{1}\left(j\right)=w\cdot F_{1}(y_{0}=start,y_{1}=j,x)\;j=1,2,\cdots m,$$

(2) Recursion: Iterate through each state from front to back, find the maximum value of the non-normalized probability of each state l=1,2,⋯,m at position i=2,3,⋯,n, and record the state sequence label $\Psi_{i}\left(l\right)$ with the highest probability.

(12) $$\delta_{i}\left(l\right)=\underset{1\leq j\leq m}{max}\left\{\delta_{i-1}\left(j\right)+w\cdot F_{i}\;\left(y_{i-1}=j,y_{i}=l,x\right)\right\}\;l=1,2,\cdots m,$$

(13) $$\Psi_{i}\left(l\right)=\underset{1\leq j\leq m}{argmax}\left\{\delta_{i-1}\left(j\right)+w\cdot F_{i}\;\left(y_{i-1}=j,y_{i}=l,x\right)\right\}\;l=1,2,\cdots m,$$

(3) When i=n, we obtain the maximum value of the non-normalized probability and the terminal of the optimal state points sequence.

(14) $$\underset{y}{max}\left(w\cdot F\left(y,x\right)\right)=\underset{1\leq j\leq m}{max}\delta_{n}\left(j\right),$$

(15) $$y_{n}^{*}=\underset{1\leq j\leq m}{argmax}\delta_{n}\left(j\right),$$

(4) Calculate the final state points output sequence.

(16) $$y_{i}^{*}=\Psi_{i+1}\left(y_{i+1}^{*}\right)i=n-1,n-2,\cdots ,1,$$

(5) Finally, the optimal sequence of state points is as follows:

(17) $$y^{*}={\left(y_{1}^{*},y_{2}^{*},\cdots ,y_{n}^{*}\right)}^{T}.$$

## 4. Experimental Results and Analysis

### 4.1. Implementation Details

**Figure 6:**
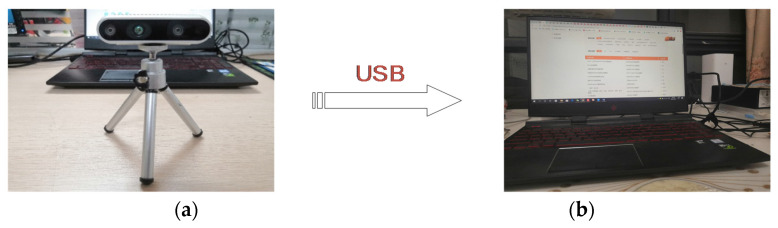
Hardware connection diagram: (**a**) Intel RealSense D435i camera; (**b**) Hewlett-Packard OMEN 4 Laptop.

In order to verify the performance of our algorithm, we carried out experimental verification at the Science Building of Beijing University of Technology. The hardware we use includes an Intel RealSense D435i camera and a computer. The diagram is shown in Figure 6. The camera is internally integrated with a Bosch BMI055 six-axis inertial sensor, so it can be used to collect image information and IMU information. The online temporal calibration method is used to synchronize the time between the camera and the IMU to ensure that the data collected by the two can correspond [31]. The frame rate of the camera is set to 30 fps, and the frequency of the IMU is set to 650 Hz. The camera transmits data to the computer via a Universal Serial Bus, and then carries out the VIO algorithm and map matching algorithm in the computer, and finally outputs the optimal trajectory of the pedestrian. The model of the computer we use is Hewlett-Packard OMEN 4 (the processor of the computer is Intel Core i5-8300H). All software is implemented in C++/MATLAB language.

### 4.2. Two-Dimensional Map Matching Experiment

**Figure 7:**
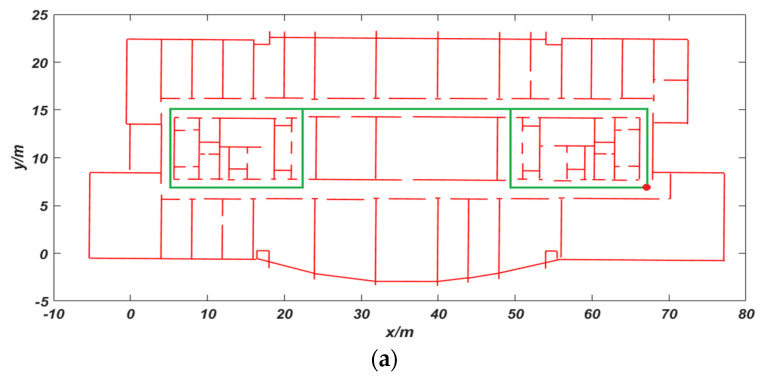

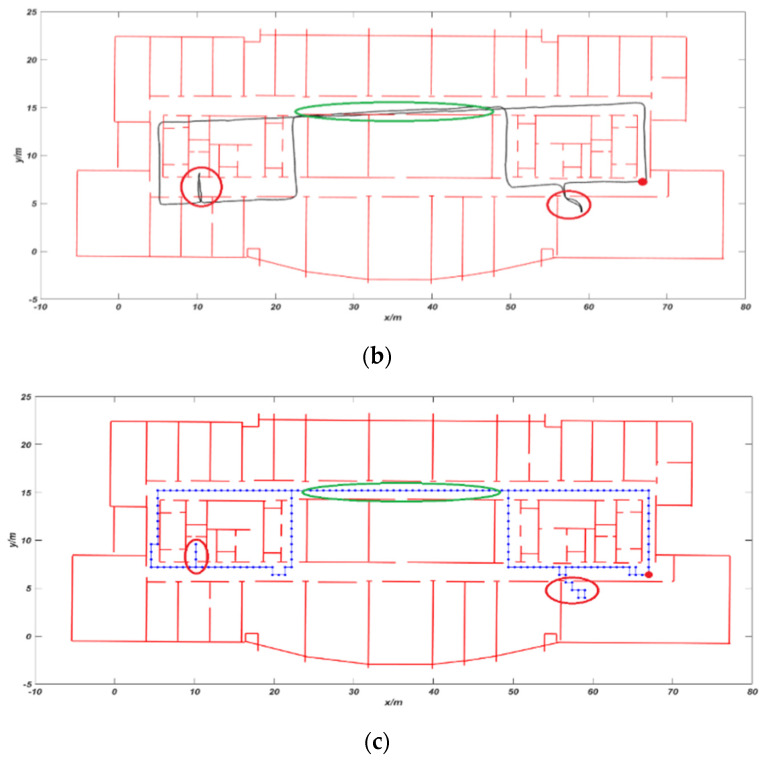
Comparison of trajectory: (**a**) preset trajectory; (**b**) VIO trajectory; and (**c**) trajectory using the conditional random field (CRF) algorithm.

The first and second experiments were performed on the ninth floor of Science Building at Beijing University of Technology. The tester is a woman with a height of 1.63 m. She started from the red dot position, walked around the trajectory as shown in the Figure 7a, and eventually returned to the starting position. The average walking speed is 0.8 m/s. In Figure 7b, the black trajectory is the positioning trajectory of VIO. The red circle area is to enter the room. It can be seen that the trajectory of VIO is accurate at first, but the positioning accuracy of the green circle in the figure starts to decrease. After that, the pedestrian trajectory showed the serious phenomenon of passing through the wall. The main reason for the failure in positioning is that the surrounding environment of the aisle here is dominated by white walls and lacks visual valid texture information. The total distance of the experiment is 156 m, the mismatch rate reached 12.8%, and the cumulative error reached 4.26 m. The cumulative error is the Euclidean distance between the preset end point and the actual end point. The mismatch rate is the ratio between the mismatched state point and the actual total matched state point in the whole walking process. The blue trajectory in the Figure 7c is the VIO trajectory after using CRF feedback. The mismatch rate was reduced to 2.3% and the cumulative error was reduced to 0.61 m. The modified trajectory is more consistent with the indoor movement of pedestrians and basically corrects the phenomenon of the trajectory passing through the wall. 

**Figure 8:**
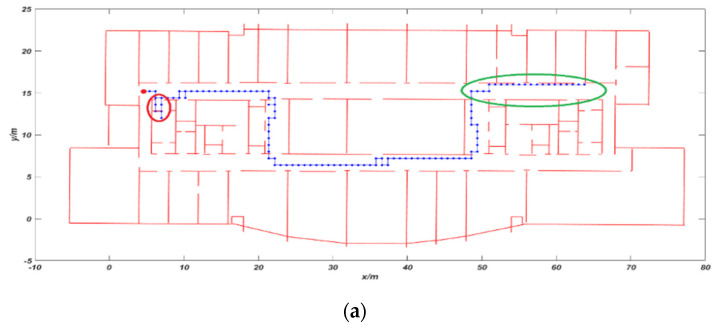

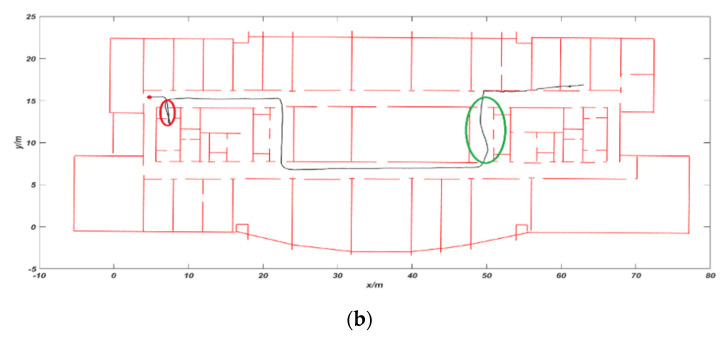
Comparison of trajectory: (**a**) VIO trajectory and (**b**) trajectory using CRF algorithm.

The second experiment is to verify the effect on the area with poor light. The tester is a man with a height of 1.78 m and the average walking speed is 1.2 m/s. As shown in Figure 8, the red dot is used as the starting position, the red circle area is entering the room and the green circle area will dim indoor lights. In Figure 8a, the VIO trajectory began to drift in the green area, the positioning error gradually increased, and eventually passed through the wall. At this time, we cannot correctly locate the tester. The total distance of the experiment is 102.8 m, the cumulative error is 2.62 m, and the mismatch rate reached 8%. The blue trajectory in Figure 8b is the VIO trajectory after using CRF feedback. It can be seen that our algorithm can solve the phenomenon of passing through walls and the accuracy of VIO trajectory has been significantly improved. The mismatch rate was reduced to 2% and the cumulative error was reduced to 0.43 m.

### 4.3. Three-Dimensional Map Matching Experiment

**Figure 9:**
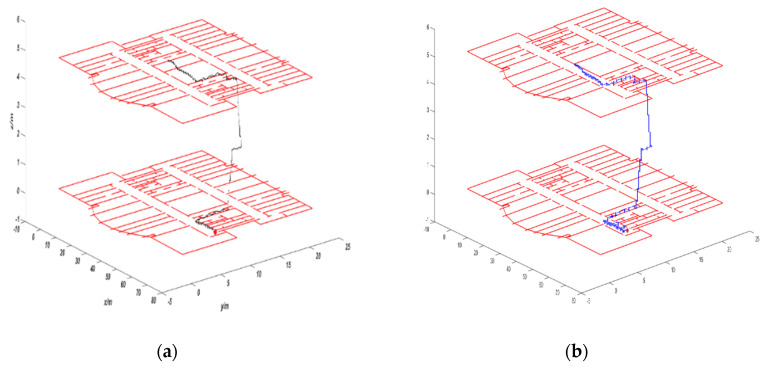
Comparison of trajectory: (**a**) VIO trajectory and (**b**) trajectory using CRF algorithm.

The third experiment was carried out on the ninth and 10th floors of Science Building in Beijing University of Technology. The tester is a man with a height of 1.7 5 m and the average walking speed is 1.0 m/s. The tester starts from the red dot on the ninth floor and go up the stairs to the 10th floor. The total distance is 59.2 m. Figure 9a is the trajectory of VIO output. As can be seen from the figure, after the floor changes, the height values of VIO trajectory have obvious drift phenomenon. The main reason is that the surrounding environment of the stair is mainly white walls, the texture information is relatively missing, and the maximum height error reaches 1.32 m. Figure 9b is the VIO trajectory corrected using the CRF model. The trajectory of the corrected VIO is significantly improved, and the cumulative height error is reduced to 0.2 6 m. Compared with the map matching algorithm in the study [25], we added the data of camera, so we achieved better accuracy. In study [26], the experiment was only carried out in the corridor and did not enter the room. In the study [27], the experiment was only performed on the same floor, and the 3D map matching is not realized.

## 5. Conclusions

In this paper, in order to improve VIO positioning accuracy in indoor environments where insufficient light and featureless, we propose an algorithm based on the combination of VIO system and 3D map matching for indoor positioning. By establishing a conditional random field model, the values output by the VIO system are input into the CRF model as observation information, and the optimal state points sequence is solved. This is used as feedback value to correct the trajectory of the VIO to improve the accuracy of indoor positioning. We conducted experiments in the indoor areas with insufficient light and lack of texture, and finally verified the effectiveness of our algorithm. Our algorithm is not only applicable to the VIO system used in this paper, but also to other VIO system. Although the algorithm proposed in this paper depends on indoor map, it has some limitations. However, it provides an effective way to improve the accuracy of VIO indoor positioning. In order to make the proposed algorithm more general and practical, the future work will be focus on map construction.

## Figures and Tables

**Table 1:** Match selection based on heading.

Range of Heading	States Choice
0~π/2	1
π/2~π	2
π~3π/2	3
3π/2~2π	4
